# In memory of Pierre Chambon (1931–2026): a visionary pioneer of modern molecular biology

**DOI:** 10.1038/s44318-026-00840-x

**Published:** 2026-06-24

**Authors:** Hinrich Gronemeyer

**Affiliations:** 1 Department of Functional Genomics and Cancer, Institut de Génétique et de Biologie Moléculaire et Cellulaire (IGBMC), Illkirch, France; 2 Centre National de la Recherche Scientifique (UMR 7104), Illkirch, France; 3 Institut National de la Santé et de la Recherche Médicale (U 1258), Illkirch, France; 4 Université de Strasbourg, Illkirch, France

On May 5, 2026, Europe—and indeed the world—lost one of the giants of molecular biology and genetics. Pierre Chambon was a visionary scientist and charismatic leader whose discoveries fundamentally transformed our understanding of eukaryotic gene organization, chromatin structure, and transcriptional regulation.

**Figure:**
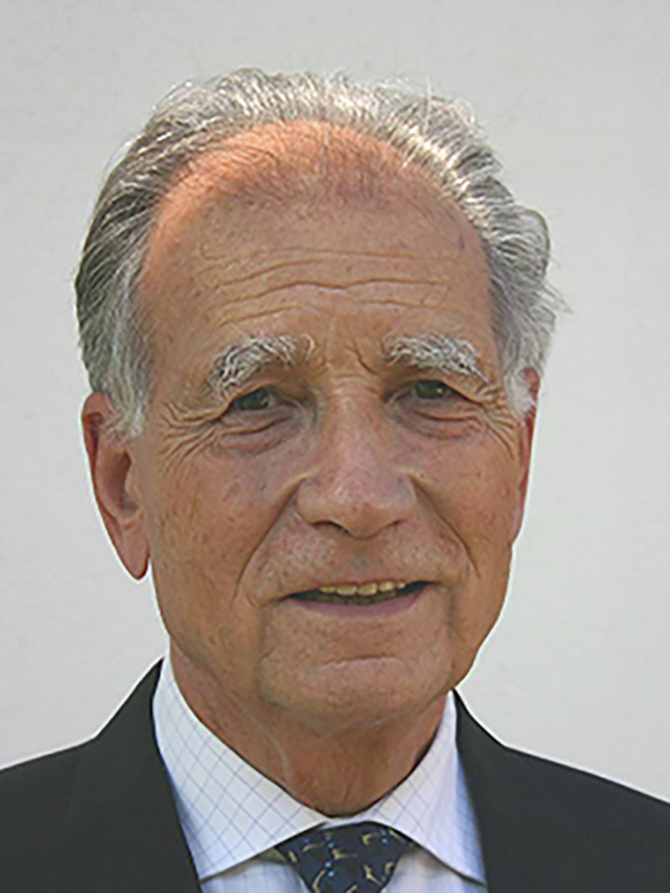
Pierre Chambon/Photograph courtesy of Brigitte Chambon

His ability to identify and tackle the most important biological questions of his time was extraordinary. As an Associate Professor at the Faculty of Medicine of the University of Strasbourg (then Université Louis Pasteur), he discovered a nuclear enzyme responsible for the synthesis of poly(ADP-ribose) (Chambon et al, 1963). Unbeknownst to him at the time, inhibitors of this enzyme, later known as PARP inhibitors, would decades later become the first approved cancer therapeutics to specifically target defects in the DNA damage response, revolutionizing the treatment of BRCA1/2-mutated breast and ovarian cancers, among others.

Following a research stay in the laboratory of Nobel laureate Arthur Kornberg, Chambon recognized the transformative potential of RNA polymerases as central regulators of gene expression. He went on to demonstrate the existence of distinct RNA polymerases (Kedinger et al, 1970), independently and almost contemporaneously with Robert Roeder and William Rutter, whose closely related findings appeared only shortly before. These discoveries laid essential foundations for the modern understanding of eukaryotic transcription.

Although this is sometimes no longer fully appreciated by younger generations of scientists, Pierre Chambon’s subsequent contributions became foundational and have long entered into textbooks: Having established the necessary experimental infrastructure together with Pierre Oudet, he demonstrated based on biochemical and electron microscopy data that chromatin is organized as a flexible chain of repeating spherical particles, for which he coined the term “nucleosomes”—a concept now universally adopted (Oudet et al, 1975). In 1977, he showed that the ovalbumin gene is composed of discontinuous segments (Breathnach et al, 1977), with the mature mRNA assembled through their precise joining. One year later, his team formulated “Chambon’s rule,” describing the conserved excision–ligation boundaries at intron–exon junctions (Breathnach et al, 1978).

In the following years, Pierre’s group developed and extensively applied in vitro transcription systems to define the sequence requirements for transcriptional initiation (Benoist and Chambon, 1981; Corden et al, 1980; Mathis and Chambon, 1981; Wasylyk et al, 1980). These studies not only dissected promoter architecture but also provided early evidence for distal regulatory elements controlling transcription, thereby helping to lay the conceptual foundations for what would soon be defined as enhancers.

When I joined Pierre Chambon’s team in 1980, he had already demonstrated an exceptional vision not only in science but also in building what would become one of Europe’s most influential research environments. Achieving this outside Paris was particularly remarkable in a country long characterized by strong centralization, and it underscored the strength of his leadership and personality.

Pierre was an extraordinarily inspiring figure. Several of his early colleagues—including Jean-Louis Mandel, Jean-Marc Egly, Christophe Benoist, and Diane Mathis—went on to establish their own highly successful research programs, collectively laying the foundations for a dynamic and enduring institutional structure. An essential contributor to this success was his wife, Brigitte Chambon, whose dedication, judgment, and organizational talent helped ensure the smooth functioning of an increasingly large and complex research enterprise.

Pierre’s next major scientific breakthrough was already foreshadowed by his longstanding fascination with steroid hormone signaling and transcriptional regulation. What was still missing were the key molecular mediators—the hormone receptors themselves. In 1986, his group reported the cloning of the estrogen receptor gene (Green et al, 1986), followed six months later by that of the progesterone receptor (Jeltsch et al, 1986). Comparison of the human and chicken estrogen receptor sequences provided the first glimpse of the receptor’s modular domain organization, which was subsequently validated by detailed structure–function analyses that identified the DNA-binding and ligand-binding domains (Kumar et al, 1987)—concepts that are now textbook knowledge.

These seminal discoveries were followed by a series of studies establishing nuclear receptors as ligand-regulated transcription factors and elucidating how they recognize specific hormone-response elements, acting as enhancers in the vicinity of their target genes. A year later, Pierre’s laboratory demonstrated that the receptor for the morphogen retinoic acid belongs to the nuclear receptor superfamily (Petkovich et al, 1987), a finding that led to the identification of additional retinoic acid receptors and their isoforms.

This was an extraordinarily productive period for the laboratory. I vividly remember Pierre showing me a *Science Watch* ranking of institutions in molecular biology and genetics based on citation impact between 1988 and 1992. His institute was ranked sixth worldwide—ahead of MIT, Princeton, Rockefeller, and Harvard—an achievement of which he was justifiably proud.

Pierre did not hesitate to transfer scientific knowledge to industry, as evidenced by collaborations with French and German pharmaceutical companies. His entrepreneurial spirit was apparent early on: in 1979, he co-founded Transgene S.A., one of the first biotechnology companies in France, with the aim of harnessing the emerging tools of genetic engineering for biomedical applications.

A major turning point came in July 1989 with the signing of a long-term partnership between the institute and the American pharmaceutical company Bristol-Myers Squibb. This visionary alliance provided the resources for the construction of a completely new research center south of Strasbourg—the Institut de Génétique et de Biologie Moléculaire et Cellulaire (IGBMC). In addition to Pierre’s own teams, the new institute included the structural-biology group of Dino Moras, creating a unique environment that combined molecular genetics, cell biology, and structural biology. Inaugurated in 1994, the IGBMC rapidly became one of Europe’s leading centers for life science research and a lasting testament to Pierre’s scientific ambition and organizational talent.

The creation of the IGBMC generated enormous enthusiasm and scientific momentum. The integration of structural biology expertise led to a series of landmark discoveries, including the first crystal structures of the ligand-binding domains of retinoid and rexinoid receptors. These studies culminated in the definition of the canonical architecture of the ligand-binding domain shared by nuclear receptors (Wurtz et al, 1996), providing a structural framework that transformed our understanding of this important family of transcription factors.

Although the institute became increasingly diversified—its research activities now span six major areas of the life sciences—Pierre continued to pursue new ambitions. Building on pioneering advances in mouse genetics, particularly the development of conditional and tissue-specific somatic mutagenesis (Feil et al, 1996), he established a dedicated mouse genetics center, the Institut Clinique de la Souris (ICS). Countless important discoveries have emerged from the IGBMC and the ICS, and their success will remain inseparably linked to Pierre’s vision, leadership, and scientific creativity.

Pierre Chambon’s scientific accomplishments were recognized worldwide through numerous distinctions and awards, notably the CNRS Gold Medal (1979), the Albert Lasker Award for Basic Medical Research (2004), and the Canada Gairdner International Award (2010). Pierre was elected an *EMBO Member* already back in 1975, and served as EMBO’s Secretary General from 1990 to 1995. He was elected to the *Académie des sciences* and as a Foreign Associate of the National Academy of Sciences of the USA in 1985, and as a Foreign Member of the Royal Swedish Academy of Sciences in 1987. He held the Chair of Molecular Genetics at the *Collège de France* from 1993 to 2002. France honored him with two of its highest distinctions, appointing him Commander of the *Légion d’honneur* and Grand Officer of the *Ordre national du Mérite*.

My colleagues and I will remember Pierre as a demanding yet inspiring leader, a visionary scientist, and a charismatic personality. He possessed an extraordinary memory, an exceptionally sharp intellect, and an unwavering dedication to science that kept him intellectually engaged well beyond the age of 90. Outside the laboratory, he enjoyed skiing, scuba diving, and sharing good meals with friends and colleagues, pursuits he embraced with the same enthusiasm that he brought to science. We will miss him deeply and remember him with great gratitude and affection.
